# Structural polymorphism of the low-complexity C-terminal domain of TDP-43 amyloid aggregates revealed by solid-state NMR

**DOI:** 10.3389/fmolb.2023.1148302

**Published:** 2023-03-29

**Authors:** Jayakrishna Shenoy, Alons Lends, Mélanie Berbon, Muhammed Bilal, Nadia El Mammeri, Mathilde Bertoni, Ahmad Saad, Estelle Morvan, Axelle Grélard, Sophie Lecomte, François-Xavier Theillet, Alexander K. Buell, Brice Kauffmann, Birgit Habenstein, Antoine Loquet

**Affiliations:** ^1^ University Bordeaux, CNRS, Bordeaux INP, CBMN, UMR 5248, IECB, Pessac, France; ^2^ University Bordeaux, CNRS, INSERM, IECB, UAR 3033, Pessac, France; ^3^ Institute for Integrative Biology of the Cell (I2BC), CEA, CNRS, Université Paris-Sud, Université Paris-Saclay, Gif-surYvette Cedex, France; ^4^ Department of Biotechnology and Biomedicine, Technical University of Denmark, Lyngby, Denmark

**Keywords:** TDP-43, amyotrophic lateral sclerosis, frontotemporal dementia, amyloid, polymorphism, solid-state NMR, low-complexity domain

## Abstract

Aberrant aggregation of the transactive response DNA-binding protein (TDP-43) is associated with several lethal neurodegenerative diseases, including amyotrophic lateral sclerosis and frontotemporal dementia. Cytoplasmic neuronal inclusions of TDP-43 are enriched in various fragments of the low-complexity C-terminal domain and are associated with different neurotoxicity. Here we dissect the structural basis of TDP-43 polymorphism using magic-angle spinning solid-state NMR spectroscopy in combination with electron microscopy and Fourier-transform infrared spectroscopy. We demonstrate that various low-complexity C-terminal fragments, namely TDP-13 (TDP-43_300–414_), TDP-11 (TDP-43_300–399_), and TDP-10 (TDP-43_314–414_), adopt distinct polymorphic structures in their amyloid fibrillar state. Our work demonstrates that the removal of less than 10% of the low-complexity sequence at N- and C-termini generates amyloid fibrils with comparable macroscopic features but different local structural arrangement. It highlights that the assembly mechanism of TDP-43, in addition to the aggregation of the hydrophobic region, is also driven by complex interactions involving low-complexity aggregation-prone segments that are a potential source of structural polymorphism.

## 1 Introduction

Misfolding and deposition of proteins into fibrillar aggregates are causative of many neurodegenerative diseases, including Alzheimer’s and Parkinson’s disease, and many other cognitive disorders (Selkoe and Hardy, 2016; Chiti and Dobson, 2017; Eisenberg and Sawaya, 2017; Peng et al., 2020). These proteinaceous aggregates consist generally of amyloid fibrils, and they usually display a typical X-ray diffraction (XRD) pattern indicative of cross-β structure, irrespective of their primary sequence (Sunde et al., 1997). Amyloid deposits often exhibit a fibrillar morphology, they are rich in β−sheet secondary structure elements, and they bind to organic dyes such as thioflavin-T (ThT), congo red, or its derivatives (Dapson, 2018; Aliyan et al., 2019; Lee et al., 2019).

TDP-43 is an essential human protein of 414 amino acids encoded by the *TARDBP* gene and widely expressed in neurons. The protein binds to DNA and RNA (Tollervey et al., 2011) and is involved in post-transcriptional RNA processing and nuclear body formation (Ou et al., 1995; Buratti and Baralle, 2001). TDP-43 is a major component of neuronal inclusions characteristic of amyotrophic lateral sclerosis (ALS) and frontotemporal lobar dementia (FTLD), and many other neurodegenerative diseases collectively called TDP-43 proteinopathies (Neumann et al., 2006; Gao et al., 2017; Nelson et al., 2019; Tziortzouda et al., 2021). TDP-43 has received much interest in the past decade because its aberrant aggregation directly affects mRNA splicing of various essential genes like CFTR (Cystic fibrosis transmembrane conductance regulator), TARDBP (TDP-43), FUS, SNCA (α-synuclein), HTT (Huntingtin), and APP (Amyloid precursor protein, amyloid-β) (Nakashima-Yasuda et al., 2007; Johnson et al., 2011; Josephs et al., 2015; Uchino et al., 2015). The aggregation process has been associated with several incurable diseases, including cystic fibrosis, ALS, FTLD, Alzheimer’s, Parkinson’s, and Huntington’s disease (Buratti and Baralle, 2001; Schwab et al., 2008; Polymenidou et al., 2011; Sephton et al., 2011; Polymenidou et al., 2012). ALS is a progressive motor neuron degenerative disease-causing muscle atrophy, and FTLD deteriorates neurons in the brain’s frontotemporal lobes, leading to dementia and cognitive impairment. About 90% of ALS patients and about 50% of FTLD patients are observed with ubiquitinated TDP-43 neuronal inclusion (Ling et al., 2013; Tan et al., 2017).

**FIGURE 1:**
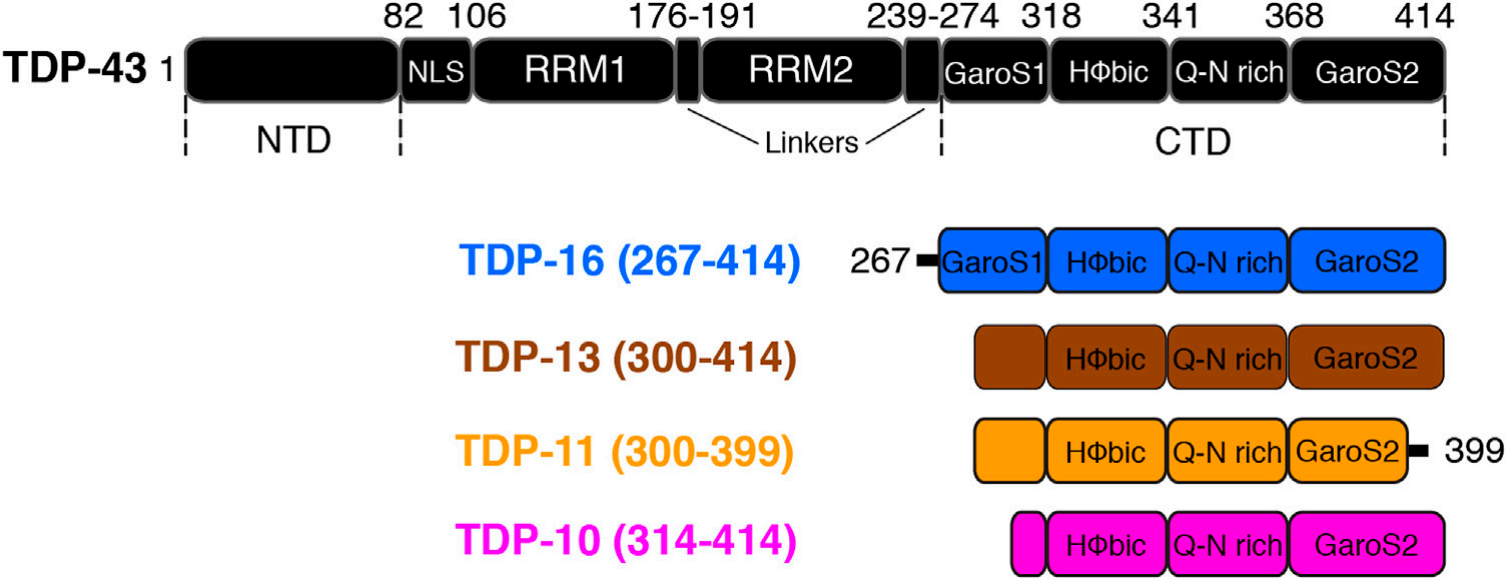
Sequences of TDP-43 and various low-complexity C-terminal domain fragments used in this study. Boxes are used to denote different functional/structural domains. The nomenclature of the different CTD fragments is chosen to be consistent with (Mompean et al., 2016b; Shenoy et al., 2019).

TDP-43 is composed of a well-folded N-terminal domain (NTD, amino acid (aa) 1–81) (Chang et al., 2012; Qin et al., 2014; Mompean et al., 2016a; Tsoi et al., 2017), a nuclear localization signal (NLS, aa 82–106), two highly conserved RNA recognition motifs RRM1 (aa 106–175) and RRM2 (aa 192–262) that binds UG rich RNA (Ayala et al., 2005; Lukavsky et al., 2013; Pillai and Jha, 2018), a nuclear export signal (NES, aa 239–274) and a C-terminal domain (CTD) (aa 275–414). The CTD comprises one hydrophobic segment (318–340), a Q/N-rich segment (341–367) that is flanked by two subdomains rich in glycines, serines, and aromatic residues called GaroS1 (267–318) and GaroS2 (368–414) (Mompean et al., 2016b) (Figure 1). The TDP-43 CTD is essential for RNA splicing activity and interaction with several partner proteins (Ayala et al., 2005; Buratti et al., 2005; Freibaum et al., 2010; Conicella et al., 2016). Due to its typical amino acid composition rich in polar residues and poor in aliphatic residues, the CTD has been classified as a low-complexity domain (LCD) (March et al., 2016; Guenther et al., 2018) and it has been associated with prion-like properties (Cushman et al., 2010; Fuentealba et al., 2010). Like many other reported prion-like domains, the TDP-43 CTD is intrinsically disordered and highly aggregation-prone, playing a critical role in TDP-43 aggregation (Santamaria et al., 2017), and it is sufficient to form amyloid aggregates (Cushman et al., 2010; Guo et al., 2011; Jiang et al., 2013; Mompeán et al., 2014; Budini et al., 2015). Interestingly, 90% of disease-related mutations and many post-translation modification sites are located at the CTD (Kwon et al., 2012; Buratti, 2015). TDP-43 CTD is also necessary and sufficient for liquid droplet formation *via* transient helix formation by the hydrophobic subregion (320–340) essential for TDP-43 function, and disruption of these interactions contributes to TDP-43 dysfunction and pathology (Conicella et al., 2016; Conicella Alexander et al., 2020). Recently, TDP-43 proteinopathies and synucleinopathies have been linked based on *in vitro* interaction between TDP-43 and α-synuclein (Dhakal et al., 2021; Dhakal et al., 2022).

High-resolution structures of TDP-43 fibrils obtained after a sarkosyl-based chemical extraction from two ALS patient brains have been reported (Arseni et al., 2022). Both structures display a double-spiral-shaped fold spanning residues 282 to 360. Recently, fibrils formed by the CTD of the transmembrane protein 106B (TMEM106B) have been identified in *postmortem* brain tissue of FTLD patients (Jiang et al., 2022; Schweighauser et al., 2022), raising questions as to the interplay between TMEM106B and TDP-43 (Chang et al., 2022). Interestingly, although TDP-43 was detected in the brain-extracted samples used in these studies, the protein was found to be present in the form of amorphous aggregates, preventing structure determination by cryo-electron microscopy (cryo-EM).

Indeed, due to their tendency to form insoluble nano-scale objects, amyloid fibrils still constitute challenging targets for structural biology and biophysical techniques. Recent developments in cryo-EM (Fitzpatrick and Saibil, 2019; Fändrich and Schmidt, 2021; Ragonis-Bachar and Landau, 2021), together with advanced magic-angle spinning solid-state NMR techniques (MAS ssNMR) (Petkova et al., 2002; Wasmer et al., 2008; Colvin et al., 2016; Tuttle et al., 2016; Mompean et al., 2018; Gelenter et al., 2019; Daskalov et al., 2021; Dregni et al., 2022) offer robust and powerful approaches to tackle the structural conformation of amyloid fibrils at the atomic level. Pathological amyloid fibrils arising from misfolding events are prone to display structural polymorphism, a phenomenon in which polypeptides with highly similar sequences under the same or different environmental conditions (e.g., protein concentration, buffer composition, incubation temperature, pH, *etc.*) adopt multiple structurally distinct molecular conformations (Tycko and Wickner, 2013; Gallardo et al., 2020; Lutter et al., 2021). Different polymorphs may show a difference in fibril diameter, fibril morphology (straight or twisted), the number of protofilaments per bundle, or at the level of the tertiary fold. It is essential to study and understand structural polymorphism in pathological amyloids as a distinct structural polymorph of the same amyloid protein can display variation at the level of clinical and neuropathological features and cytotoxicity (Petkova et al., 2005; Qiang et al., 2017; Goedert et al., 2018). Accumulating data on amyloid-β, tau, and α-synuclein protein aggregates displaying various polymorphs associated with specific disease subtypes and self-propagating ability reinforces the notion of distinct “structural strains” (Aguzzi et al., 2007; Eisenberg and Sawaya, 2017). Interestingly, the sarkosyl-insoluble TDP-43 fraction derived from different FTLD subtypes indicates a difference at the level of aggregate morphologies, sensitivities to proteolysis, as well as neurotoxicity (Tsuji et al., 2012; Laferriere et al., 2019). The peptides derived from RRM2 ^247^DLIIKGISVHI^257^ have been shown to adopt multiple amyloid polymorphs characterized by the difference in backbone conformation and symmetry classes of steric zippers (Guenther et al., 2018).

In a previous study (Shenoy et al., 2019), we have shown using MAS ssNMR and other biophysical techniques that *in vitro* aggregates obtained at pH = 7.5 of various TDP-43 fragments do not share the same structural characteristics. In particular, we have demonstrated that the CTD could adopt distinct conformations in isolation or in the presence of other domains by comparing the conformation of TDP-16 (TDP-43_267–414_) to TDP-35 (TDP-43_90–414_) and full-length TDP-43. We used a construct lacking the GaroS2 domain (i.e., TDP-43 ∆GaroS2 (TDP-43_1–366_)) to demonstrate that although GaroS2 is critical for the formation of amyloid fibrils in the full-length construct, its removal still results in the formation of fibrillar aggregates with cross-β properties (Shenoy et al., 2019). In another study, Mompean and coworkers reported distinct MAS ssNMR signatures for fibrils also obtained *in vitro* but using a different aggregation procedure (Pantoja-Uceda et al., 2021). Zhuo et al. (Zhuo et al., 2020) and Fonda et al. (Fonda et al., 2021) reported MAS NMR data on *in vitro* aggregated TDP-43 (311–360) and TDP-43 (262–414), respectively. By comparing the NMR chemical shift signature of these various TDP-43 fibrillar polymorphs, as well as to those formed after seeding with proteins from bacterial inclusion bodies (Chang et al., 2020), no consensus on the conformation of the amyloid core could be drawn (Shenoy et al., 2019; Fonda et al., 2021), highlighting the propensity of TDP-43 to adopt distinct polymorphic structure depending on the aggregation conditions and the sequence of the particular construct under study. It is interesting to note that neuronal inclusions are composed of ubiquitinated and phosphorylated aggregates of truncated TDP-43 C-terminal fragments of size ∼25–35 kDa (e.g., TDP-25 and TDP-35), formed by aberrant cleavage events and shown to be highly cytotoxic and contribute to ALS (Neumann et al., 2006; Zhang et al., 2007; Zhang et al., 2009). TDP-25 can cause neurodegeneration in mice (Walker et al., 2015), and these fragments are observed as amorphous aggregates in inclusions by cryo-electron tomography (Riemenschneider et al., 2022). Together, these observations suggest that the understanding of TDP-43 aggregation should not only be limited to structural investigation of full-length TDP-43 (1–414) but also take into account the polymorphic and conformational properties of its CTD fragments, including the low-complexity domains.

In this study, we use a combination of MAS ssNMR, electron microscopy, FT-IR, and X-ray diffraction to investigate the molecular conformation and the extent of structural polymorphism of various TDP-43 low-complexity CTD fragments in their amyloid states, namely TDP-13 (TDP-43 residues 300–414), TDP-11 (TDP-43 residues 300–399) and TDP-10 (TDP-43 residues 314–414).

## 2 Results and discussion

### 2.1 Aggregation, morphology, and X-ray diffraction of TDP-43 C-terminal fragments

Accumulating pieces of evidence, together with our previous MAS ssNMR study (Shenoy et al., 2019), have suggested that CTD fragments in their amyloid form consist not only of rigid and well ordered β-sheets secondary structure elements but also a significant part of the protein sequence may be composed of less structurally ordered regions, implying the amyloid core to be shorter than the low-complexity CTD (Mompeán et al., 2014; Cao et al., 2019; Chang et al., 2020; Zhuo et al., 2020; Capitini et al., 2021). To understand the propensity of the low-complexity CTD to adopt the amyloid fold and to investigate the degree of polymorphism, we designed three TDP-43 CTD fragments, namely TDP-13 (TDP-43 residues 300–414), TDP-11 (TDP-43 residues 300–399) and TDP-10 (TDP-43 residues 314–414) **(** Figure 1 **)**. The polymorphic capability of the TDP-43 CTD amyloid fibrils has significant relevance in TDP-43 pathology and understanding its structure and function can provide insights into the underlying mechanisms of neurodegenerative diseases and guide the development of effective therapeutical strategies. The three fragments used in our study comprise the hydrophobic segment (318–340) and the Q/N rich segment (341–367) but varied in sequences from the GaroS1 and GaroS2 subdomains. The nomenclature of different C-terminal segments is chosen to be consistent with Mompean et al. and Shenoy et al. (Mompean et al., 2016b; Shenoy et al., 2019).

The C-terminal fragment (CTF) low-complexity constructs studied here have been selected to be shorter than the LCD (267–414) as defined by Eisenberg and coworkers (Guenther et al., 2018). In TDP-13, we have shortened the fragment TDP-16 by 32 amino acids to remove the first part of the GaroS1 domain. For TDP-11, we removed the last 15 amino acids compared to TDP-13; this deletion corresponds to the last part of the GaroS2 domain. TDP-11 corresponds to an extension of the TDP-43 ∆GaroS2 (1–366) construct, which we have previously investigated, towards the C-terminus (Shenoy et al., 2019). For the third construct used in this study (TDP-10), we shortened the TDP-16 sequence even further to start at residue 314. The three sequences are shown in Supplementary Figure S1.

**FIGURE 2:**
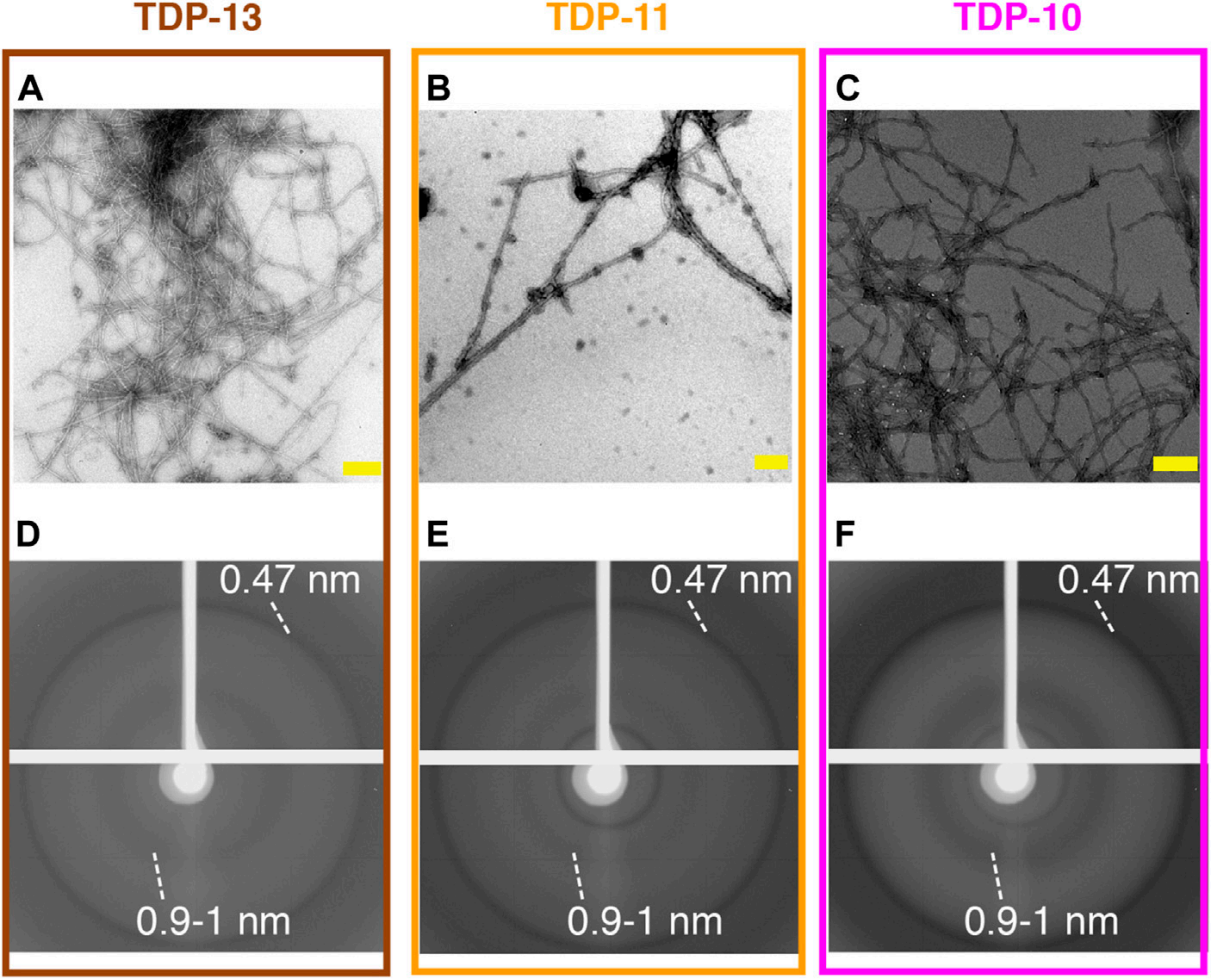
Structural analysis of low complexity CTFs aggregates. **(A–C)** Negatively stained electron micrographs of TDP-13 **(A)**, TDP-11 **(B)**, and TDP-10 **(C)** aggregates. Scale bars (in yellow) are 200 nm. **(D–F)** X-ray diffraction patterns of TDP-13 **(D)**, TDP-11 **(E)**, and TDP-10 **(F)** aggregate display characteristic cross-β reflections. Reflections at 4.7 Å and 9–10 Å are highlighted.

The three low-complexity CTF constructs were recombinantly expressed in *E. coli* and purified under denaturing conditions following the procedure reported previously (Shenoy et al., 2019). We used a 20 mM MES buffer at pH 7.5 to observe the self-assembly of purified proteins into large aggregates. To investigate the morphology of the 3 CTF aggregates, we employed negative staining electron microscopy (EM). We observed straight and unbranched filaments for the 3 CTF samples (Figures 2A–C), displaying fibril widths of ∼10–20 nm. This feature is comparable to our previous observation on TDP-43, TDP-35, and TDP-16 fibrils also obtained under *in vitro* aggregation conditions (Shenoy et al., 2019), as well as fibrillar species observed in patient brains (Hasegawa et al., 2008; Mori et al., 2008; Nonaka et al., 2013) or more recently by cryo-EM from preparations obtained after a sarkosyl-based extraction (Arseni et al., 2022). To determine the amyloid nature of the CTF aggregates from a structural point of view, we used X-ray diffraction (XRD). Amyloid fibrils displaying the canonical cross-β arrangement can be identified using XRD by the presence of a characteristic reflection ring at 0.47 nm, arising from inter-strand spacing (Sunde et al., 1997). TDP-13, TDP-11, and TDP-10 aggregates all display an intense reflection at 0.47 nm (Figures 2D–F and Supplementary Figure S2), indicating that they are structurally organized in a cross-β architecture. A similar observation was reported for TDP-43, TDP-35, and TDP-16 *in vitro* fibrils (Shenoy et al., 2019). Additional reflections are observed at ∼1 nm, assigned to intra-sheet spacing, and are commonly observed for amyloid fibril samples. Altogether, our results indicate that despite their short length compared to the full length TDP-43, CTFs as short as TDP-10 (i.e., 100 amino acids) still self-assemble into regular fibrillar assemblies and display a typical cross-β fold.

### 2.2 MAS ssNMR of CTF amyloid aggregates reveals the presence of mobile regions

**FIGURE 3:**
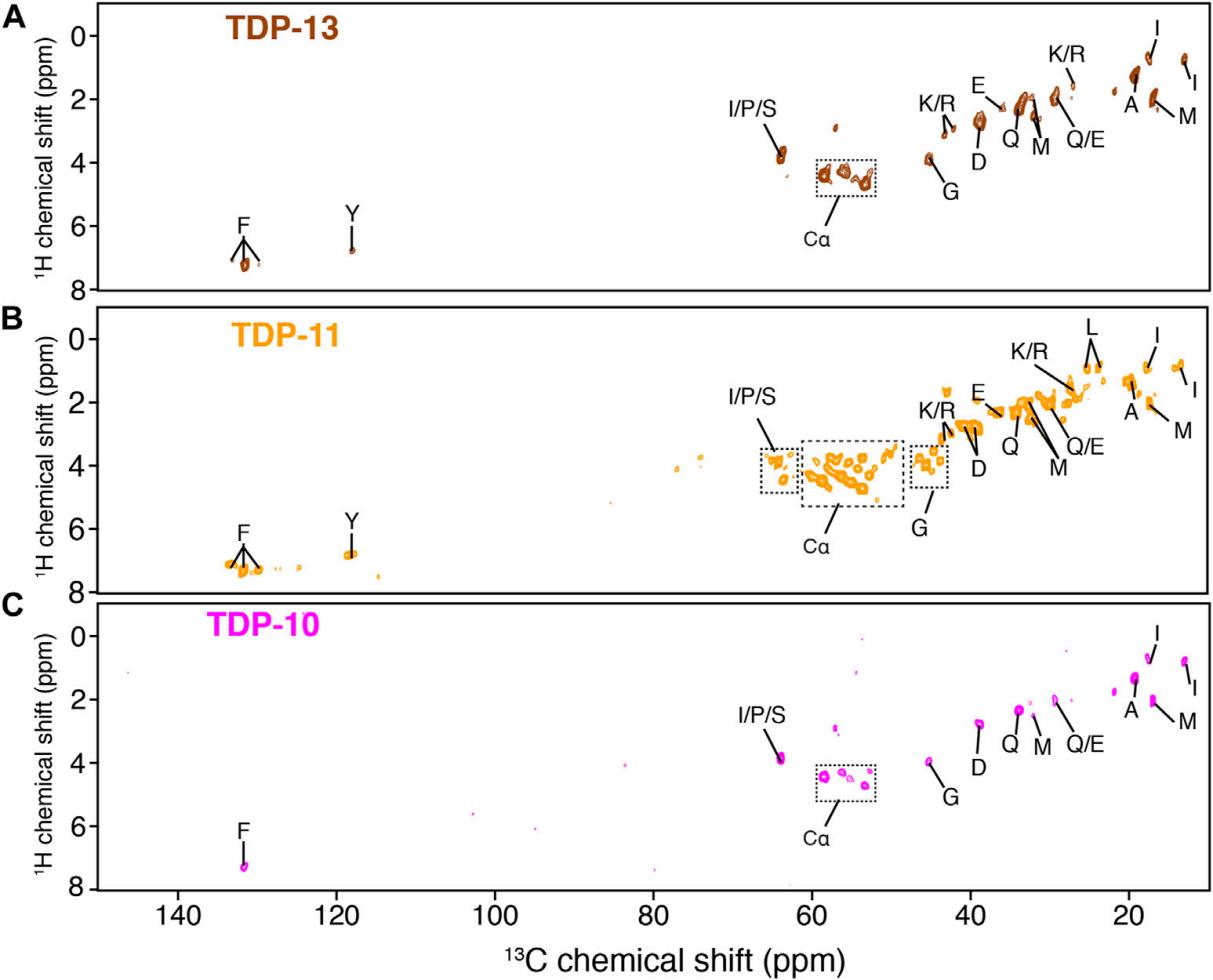
Detection of protein segments associated with high mobility by MAS NMR. ^1^H-^13^C INEPT experiments of TDP-13 (in brown) **(A)**, TDP-11 (in orange) **(B)**, and TDP-10 (in pink) **(C)** amyloid aggregates. The data were recorded at a ^1^H frequency of 600 MHz, 11 kHz MAS at 278 K.

Next, we employed MAS ssNMR to investigate the molecular organization of low-complexity CTF amyloid aggregates. In a first step, we employed J-based INEPT transfer as a starting NMR polarization transfer between ^1^H and ^13^C nuclei to select residues undergoing fast molecular movements (Matlahov and van der Wel, 2018; Siemer, 2020). Interestingly, the three CTF aggregates showed multiple signals in the 2D ^1^H-^13^C INEPT spectra (Figure 3), indicating the presence of highly mobile protein segments. The INEPT experiment can be a valuable tool to detect monomeric species in an aggregated sample, i.e., resulting from an incomplete polymerization or a monomer-fibril equilibrium. The presence of monomeric species in the NMR rotor would lead to observing all amino-acid spin systems in the primary sequence of CTFs in the INEPT experiments. The aromatic ^1^H-^13^C spectral region (i.e., 6–8 ppm for ^1^H and 120–140 ppm for ^13^C) is a good indicator because the spectral dispersion in this region allows for unambiguous identification of most aromatic contributions. Phenylalanine and tyrosine signals are observed in the INEPT spectra (Figure 3), while histidine and tryptophan are not, although in abundance in the primary sequence of CTFs. Although the absence of histidine signals could be putatively associated with a change in the local environment due to side chain protonation/deprotonation at neutral pH (Li and Hong, 2011), such deprotonation events are unlikely for tryptophan sidechain at neutral pH, suggesting that the observed signals in INEPT experiments originate from the highly mobile segments of the matured fibrillar assembly and not from non-assembled monomers.

NMR signals observed in the INEPT experiments of TDP-13 and TDP-10 aggregates are comparable; however, several new correlations are identified in the Cα region (∼54–60 ppm) of the TDP-11 construct (Hα-Cα correlation peaks are indicated with dotted squares in Figure 3), suggesting that TDP-11 aggregates contain a larger number of residues in mobile segments. Note that this observation can’t strictly rule out that TDP-10 and TDP-13 aggregates might contain fewer mobile residues in the unfavorable us-ms exchange regime compared to TDP-11. Unfortunately, only a tentative assignment of amino acid spin systems could be attempted here as INEPT signals display typically random coil conformation. The presence of mobile segments in the shorter CTFs is unexpected and suggests that the amyloid core sequence enabling aggregation might be even more concise. This inference is in line with the observation that the shorter peptide fragments, SegA (residues 311–360) and SegB (residues 286–331) from hydrophobic and Q/N rich regions, are sufficient to form amyloid aggregates. (Mompean et al., 2015; Cao et al., 2019; Zhuo et al., 2020). Moreover, Murray and coworkers have recently demonstrated that the CTD may have multiple aggregation-prone segments and that aged liquid droplets of TDP-43 adopt an amyloid core comprising the residues 365–400, which is outside of the conventional hydrophobic and Q/N rich region (311–360) essential for TDP-43 aggregation (Fonda et al., 2021). Our results suggest that the truncation of a few residues at the C- terminal extremity has an impact on the molecular organization of amyloid fibrils in TDP-43 CTF aggregates.

### 2.3 Low-complexity CTF aggregates adopt a distinct rigid polymorphic conformation

Since amyloid fibrillar architectures are known to be primarily rigid and to provide structural stability, we next characterized the molecular architecture of low-complexity CTF aggregates by taking advantage of cross-polarization (CP) techniques. CP favors the detection of rigid residues in amyloid aggregates. Hence, we employed 2D^13^C–^13^C spectroscopy with a short proton-driven spin diffusion (PDSD) mixing time of 50 ms to probe intra-residue correlations of rigid residues. The aliphatic region of the ^13^C–^13^C correlation spectra of CTF aggregates is displayed in Supplementary Figure S3. Overall, TDP-13, TDP-11, and TDP-10 CTF aggregates showed few correlations with a^13^C line width of ∼100 Hz (full width at half maximal height in the indirect dimension), indicative of the presence of residues associated with a relatively well-ordered structural arrangement within the amyloid core. Comparable ^13^C line widths have been reported for TDP-43_311–360_ by Lu and coworkers (Zhuo et al., 2020), TDP-43_274–414_ by Yang et al. (Chang et al., 2020) and for TDP-43_274–414_ by Murray and coworkers (Fonda et al., 2021). However, we also observed signals with broader line widths (∼150–250 Hz), also seen in TDP-43_274–414_ (Fonda et al., 2021), suggesting that CTF aggregates maintain a significant degree of local structural polymorphism. Finally, based on their characteristic chemical shifts and intra-residue correlation pattern, we identified serine, alanine, isoleucine, and proline residues. Also, intense peaks corresponding to glutamine and asparagine are observed in all the CTF fibrillar assemblies assigned by identifying the entire spin systems, supporting the notion of Q/N rich region involving the TDP-43 rigid amyloid core based on previous experimental and theoretical studies by Mompeán and coworkers (Mompeán et al., 2014; Mompean et al., 2015; Mompean et al., 2016b).

**FIGURE 4:**
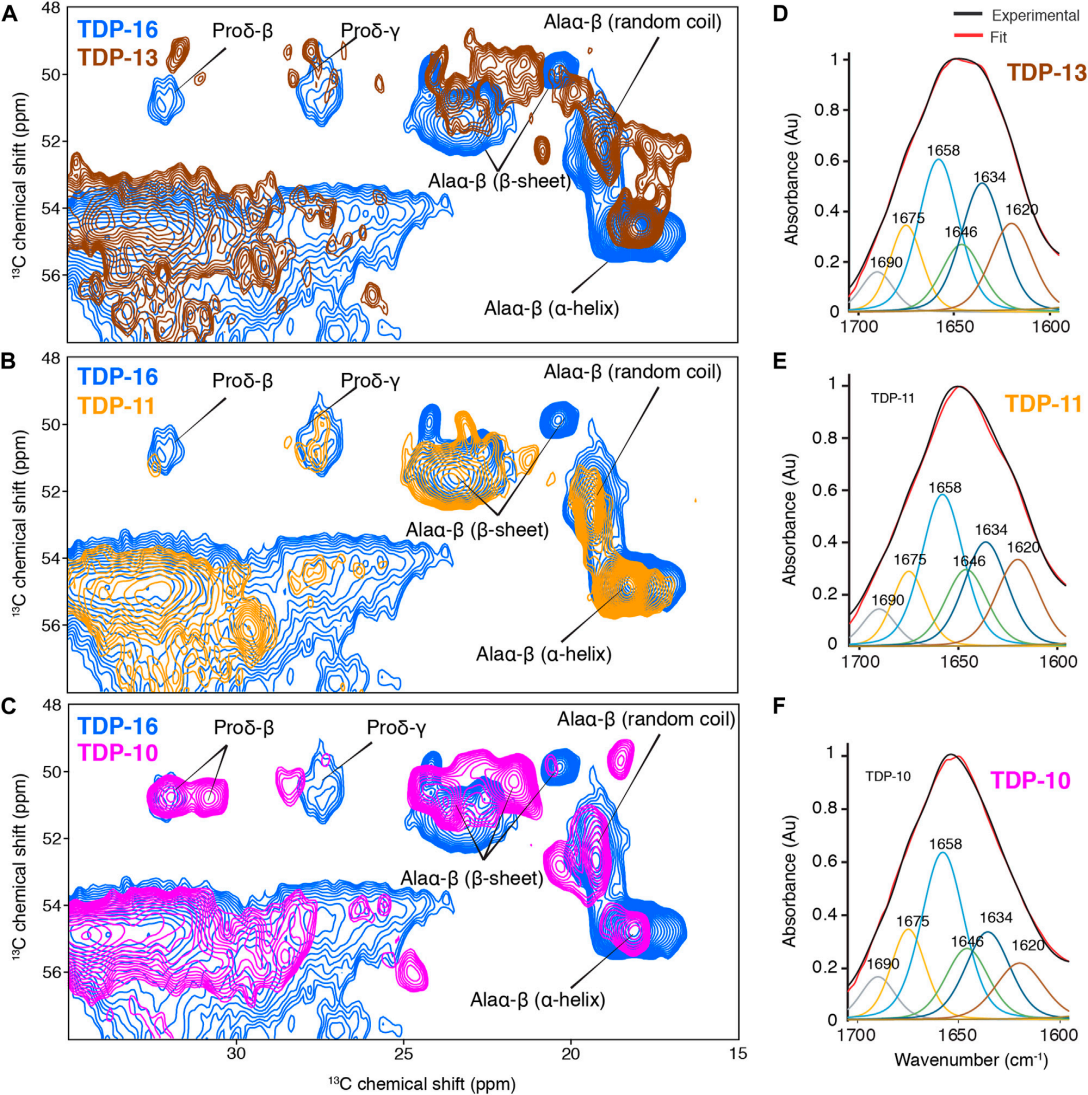
Comparison of the rigid cores of TDP-10, TDP11, and TDP-13 CTF aggregates by MAS NMR and FT-IR spectroscopy. **(A–C)** Spectral excerpts from the overlay of 2D^13^C–^13^C PDSD spectra (mixing time of 50 ms) of **(A)** TDP-13 (brown), **(B)** TDP-11 (orange), and **(C)** TDP-10 (pink) compared with TDP-16 (blue). Spectra were recorded at 600 MHz, 11 kHz MAS frequency at 278 **(K)**. Alanine Cα–Cβ and proline Cδ–Cβ correlations with chemical shift-dependent secondary structure are marked. FT-IR spectra of **(D)** TDP-13, **(E)** TDP-11, and **(F)** TDP-10 aggregates in the amide I and II range, displaying the experimental (black) and fitted curve (red). The deconvolution of amide bands are shown on the FTIR spectra, displaying contribution of secondary structure elements, namely parallel and antiparallel β-sheet: 1,634 cm^−1^ (dark blue), 1,620 cm^−1^ (brown), and 1,690 cm^−1^ (grey); α-helix: 1,658 cm^−1^ (sky blue); turns: 1,675 cm^−1^ (yellow), and random coil: 1,646 cm^−1^ (green).

To obtain information on the secondary structure content of the residues observed in the rigid amyloid cores, we performed a chemical shift analysis. ^13^C chemical shifts are sensitive probes to the local environment, and their values can be used to derive the local secondary structure (Wishart and Sykes, 1994). We compared ^13^Cα and ^13^Cβ experimental chemical shifts in CTF aggregates to the database reported by Wang and Jardetzki (Wang and Jardetzky, 2002) to extrapolate information on the secondary structure. Interestingly, the CTF fibrils are not entirely composed of β-sheets but also contain residues exhibiting chemical shifts consistent with α-helical and random coil conformations, as illustrated by proline and alanine chemical shifts (Figures 4A–C). The presence of residues that are immobilized enough to be detected in CP experiments but not involved in β-sheet secondary structure has already been unambiguously reported by us for TDP-35 fibrils (Shenoy et al., 2019), and others on fibrils aggregated from the C-terminal domain of TDP-43 (Chang et al., 2020; Fonda et al., 2021), also using ^13^Cα and ^13^Cβ chemical shift analysis.

Furthermore, we also observed that the different CTF fibrils adopted distinctive molecular architecture, as exemplified by comparing the alanine Cα-Cβ correlation region (Figures 4A–C). The comparison of their spectral fingerprint suggests that the three TDP-43 CTF fibrils display considerable variations in the number of alanine signals, their secondary structure content, and their ^13^C line width. Because the three samples were prepared using the same buffer, protonation state, and electrostatic environment, it suggests that the observation of different spectral signatures is associated with a noticeable structural polymorphism between the final CTF fibrillar structure. Considering that the three CTF constructs only differ by <10% of their primary sequence, it indicates that small changes at the level of the N-terminal and C-terminal amino acids are enough to drive the aggregation mechanisms into different fibril structure, highlighting the high propensity of TDP-43 to adopt polymorphic structures *in vitro*. Note that we observed very broad signals in the ^15^N dimension, hampering the use of further ^15^N-^13^C experiments.

We decided to further complement the MAS ssNMR chemical shift analysis with Fourier-Transform infrared (FT-IR) spectroscopy. FT-IR is a powerful method to obtain a rapid analysis of the secondary structure of peptides and proteins in their aggregated state (Zandomeneghi et al., 2004). We performed Attenuated total reflection (ATR) FT-IR spectroscopy to study the relative amount of secondary structure elements of TDP-43 CTF fibrils. TDP-13, TDP-11, and TDP-10 aggregates display comparable ATR-FTIR spectra in the amide I and II range**.** The amide I band (1,600–1,690 cm^−1^) primarily arises from backbone carbonyl stretching vibrations and is directly related to secondary structure (Byler and Susi, 1986; Krimm and Bandekar, 1986), while the amide II band (1,480–1,575 cm^−1^) corresponds to CN stretching and NH bending. ATR-FTIR analysis also evidences a composite secondary structure in TDP-43 CTF fibrils (Figures 4D–F; Table 1). The secondary structure content can be estimated from the FT-IR signal in the amide I and amide II range from a deconvolution analysis of amide band with specific marker bands for α-helix (1,658 cm^−1^), β-sheet (1,620, 1,634, and 1,690 cm^−1^), turns (1,675 cm^−1^) and random coil (1,646 cm^−1^) (Goormaghtigh et al., 1994). The three CTF constructs (TDP-13, TDP-11, and TDP-10) display a comparable amount of β-sheet secondary structure of ∼40–50% and a significant amount of non-β-sheet conformation in their fibrillar architecture, which we interpret as putative loops and protein segments that connect β-strands in the amyloid core, as well as unstructured N-terminal and C-terminal domains surrounding the amyloid core. This result is in line with the recent cryo-EM structure of *in vitro* TDP-43 LCD fibrils, with ∼40% of residues being involved in β-sheet structural elements. MAS ssNMR data acquired on *in vitro* aggregated fibrils of the CTF TDP-43_274–414_ seeded from inclusion bodies also suggested that only ∼50% of the construct contributed to the rigid amyloid core (Chang et al., 2020).

**TABLE 1:** Secondary structure content of TDP-43 CTF amyloid fibrils probed by ATR‐FTIR analysis.

		Percentage of structural elements (%)			
Secondary structure element	Wavenumbers (cm^−1^)	TDP-43	TDP-13	TDP-11	TDP-10
β-sheets//or anti//	1,620, 1,635, 1,690	52	46	42	49
Random coil	1,646	15	13	18	13
α−helices	1,658	23	28	28	26
Turn	1,675	10	13	12	12

**FIGURE 5:**
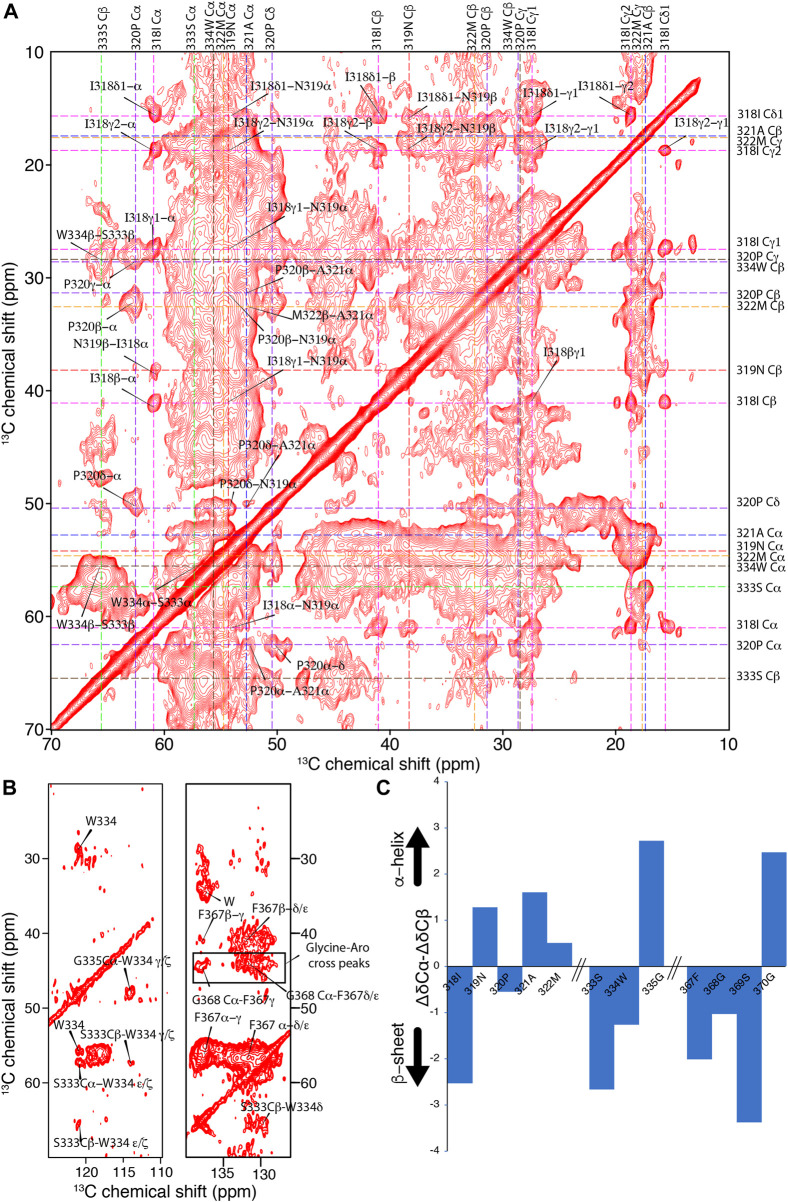
Investigation of the amyloid core of TDP-13 amyloid aggregates. **(A)** Aliphatic and **(B)** aromatic region of 2D^13^C–^13^C correlation experiment of TDP-13 amyloid aggregates recorded at a ^1^H frequency of 800 MHz at 278 K and 11 kHz MAS. A PDSD mixing time of 150 ms was used to favor sequential (i.e., residue i to i ± 1) connectivity indicated by color-coded dotted lines. **(C)** The secondary chemical shift analysis of stretches 318–322, 333–335, and 367–370 comparing experimentally obtained chemical shift with that of random-coil conformation using the equation ΔδCα–ΔδCβ (Wang and Jardetzky, 2002). Positive and negative values corresponds to α-helical or β-strand conformation, respectively.

Next, we attempted to identify amino acid segments involved in the rigid amyloid core of TDP-13 fibrils, considering its reasonably good spectral resolution. To identify these characteristic protein stretches, we conducted 2D^13^C–^13^C correlation experiments with a mixing time of 150 ms to favor the detection of sequential (*i.e.,* residue i to i±1) connectivity (Figure 5A). A sequential walk could be observed for the stretch I318-M322 because only a unique combination in the sequence leads to identifying the Ile-Asn-Pro-Ala segment. Furthermore, we assigned the stretch F367-G370 based on a single F-G-S-G correlation and S333-W334-G335 based on the single unique Ser-(aromatic residue)-Gly sequential correlations. The poor spectral resolution and sensitivity limit further unambiguous sequential assignments.

The aromatic rings of W334 and F367 are very rigid, showing strong signals in the aromatic region (∼110–140 ppm) of the 2D^13^C–^13^C correlation spectrum (Figure 5B), suggesting that these aromatic side chains could be involved in inter-residue interactions and contribute to the amyloid core stability. In addition, several unassigned correlations between glycines and aromatic residues (Tyr or Phe) can be observed in the aromatic region, suggesting they are part of the amyloid core (Figure 5B). These inter-residue correlations in the NMR spectra suggest that TDP-13 fibrils have a single dominant fibril structure, considering that unique peaks are observed. However, the broad line width observed for these cross-peaks reflects a conformational flexibility at the local level. Interestingly, the sequential correlations for the amino acid stretch I318-M322, S333-G335, and F367-G370 correspond to the hydrophobic and the GaroS2 subdomain, respectively. The secondary chemical shift analysis for these segments reveals a mixture of β-sheet and non-β-sheet conformation (Figure 5C). Unfortunately, we could not unambiguously assign the entirety of the conserved hydrophobic region (aa 320–343), that has shown to be critical for TDP-43 liquid-liquid phase separation by adopting a transient helical conformation, due to weak signals, possibly due to their dynamic nature. Non-etheless, we were able to observe that A321 and M322 adopt helical conformation, which may be extending up to the position 330, given that our data shows strong peaks corresponding to helical alanine residues that could represent the poly-alanine stretch 324–329 (5 alanines). Together with our ATR-FTIR data reporting a significant amount of helical segments (∼28%), this suggests that the helical region (321–330) is intact in TDP-13 fibrils. This observation of poly-alanine stretch (321–329) in helical-like conformation is also reported by Murray and coworkers for TDP-43 -CTD (273–414) fibrils (Fonda et al., 2021).

Interestingly, several unassigned Trp/Phe-Gly correlations can be observed in the aromatic region, suggesting they are part of the rigid amyloid core. Most of these aromatic-glycine interactions in TDP-13 belong to the GaroS2 subdomain (two Phe-Gly rich motifs at position 367–368 and 396–403, Trp-Gly motifs at position 384–386 and 411–413). This observation is in line with our previous findings that the GaroS2 domain is involved in the TDP amyloid core (Shenoy et al., 2019). Recently, Mompean *et al.* have pointed out that the interactions of the Phe-Gly motifs are a main driving force for the TDP-43 prion domain fibril formation from droplets by using solution and solid-state NMR analysis (Pantoja-Uceda et al., 2021). Interestingly, W334 is shown to be a key aromatic residue that closely interacts with the side chain carbonyls of Q343, A325, A326, A329, and S332 and contribute to TDP-43 (311–360) fibril stability (Zhuo et al., 2020). Strikingly, a mutation of W334 to Glycine disrupts phase separation without disturbing helical propensity, suggesting the importance of W334 in liquid-liquid phase separation (Li et al., 2018). Although we mainly observe residues from the hydrophobic and GaroS2 regions, we can’t rule out the possibility of a Q/N-rich region embedded in the broad signals as these residues share very close chemical shifts. Interestingly, we could not observe the segments G380-I383 and A391-G396 from the GaroS2 sub-region as in TDP-16 (267–414) from our previous study (Shenoy et al., 2019) for TDP-13 constructs. We hypothesize that this region may adopt a β-sheet conformation that is not rigid enough to be detected in the CP-based spectra or simply may not contribute to the rigid amyloid core in TDP-13 fibrils. Overall, our analysis suggests that a short and partial truncation of the GaroS1 subregion has a profound effect on the conformation of the amyloid core of TDP-13. This observation is in agreement with the notion that CTD can adopt multiple rigid fibril structures encompassing a particular region, precluding other subregions from contributing to the amyloid core (Fonda et al., 2021).

## 3 Discussion and conclusion

In the presented work, we employed a combination of solid-state NMR spectroscopy, X-ray diffraction, electron microscopy, and ATR-FTIR spectroscopy to investigate the molecular architecture and the extent of structural polymorphism of different C-terminal domain fragments of TDP-43, namely TDP-13 (300–414), TDP-11 (300–399) and TDP-10 (314–414). The presence of specific C-terminal fragments is one of the structural hallmarks of the pathology of TDP-43 proteinopathies. Interestingly, the length and the starting amino acid of the fragments seem to be controlled during the disease process, e.g., ∼18–35-kDa CTFs are found in the brains of ALS and FTLD patients (Neumann et al., 2006), while the same fragments are less abundant in the motor neurons in ALS spinal cord (Neumann et al., 2006; Igaz et al., 2008). Although these CTFs have been identified as a pathological signature of ALS and FTLD-TDP patients, their relative toxicity compared to full-length TDP-43 fibrils (Zhang et al., 2009) and their putative stronger propensity to aggregate into amyloid aggregates (Yang et al., 2010; Kasu et al., 2018) remain unclear (Berning and Walker, 2019) for *in vitro* conditions. The CTD of TDP-43 is composed of the hydrophobic domain (318–340) and the Q/N-rich domain (341–367), surrounded by two low-complexity domains (GaroS1 and GaroS2). By shortening the primary sequence of these two low-complexity domains, we generated three CTF constructs, and demonstrated that using *in vitro* conditions, already employed for full-length TDP-43 aggregation (Shenoy et al., 2019), trigger the formation of insoluble CTF aggregates. These nano-objects exhibited a typical fibrillar morphology characteristic of amyloid fibrils, and X-ray diffraction indicates the presence of a cross-β arrangement, a structural hallmark of amyloid fibrils. These findings indicate that the entire primary sequence of these low-complexity domains is not a prerequisite for amyloid fibril formation. Although the GaroS1 and GaroS2 domains are often not considered to be the main parts of the CTFs, several studies have proposed that these low-complexity domains could confer to the rigid amyloid core the ability to form hydrogels (Kato et al., 2012) by increasing the local protein concentration (Patel et al., 2015). Here we observed that the truncation of ∼30% of GaroS2 (i.e., in TDP-11), ∼50% of GaroS1 (i.e., in TDP-13), and even ∼90% of GaroS1 (i.e., in TDP-10) has little influence on the ability of the CTF to aggregate into regular amyloid fibrils. The three CTFs generate fibrils that are indistinguishable from a macroscopic point of view; however, solid-state NMR analysis revealed noticeable differences at the level of the local molecular conformation. For mobile protein segments, INEPT-based experiments showed that TDP-11 aggregates contained a higher amount of mobile residues compared to TDP-13 and TDP-10, and we hypothesize that these residues belong to the mobile flanking segments surrounding the fibrillar core. By employing CP-based experiments, we have compared the local conformation of residues involved in the rigid amyloid core. First, we observed a broad range of ^13^C line widths, as already reported by Murray and coworkers on aged liquid droplets of TDP-43 (Fonda et al., 2021), indicating that the amyloid core is partially composed of residues associated with high structural homogeneity and rigidity (narrow line width) but also by residues exhibiting important structural polymorphism (large line width), however sufficiently immobilized to be detected by CP. Although the secondary structure of residues in the amyloid core is mainly composed of β-sheets as seen by their ^13^C NMR chemical shifts, many residues harbor a non β-sheet conformation, an observation we already made for TDP-43, TDP-35, and TDP-16 in a previous study (Shenoy et al., 2019). Such observation of a non β-sheet conformation has already been reported for full-length TDP-43 (Capitini et al., 2021) and TDP-43_274–414_ (Capitini et al., 2021; Fonda et al., 2021) aggregated *in vitro*, as well as extracted from bacterial inclusion bodies (Capitini et al., 2014).

The spectral resolution observed in our samples was a limiting factor to performing a *de novo* sequential resonance assignment. Nevertheless, the comparison of the Cα-Cβ region for alanine residues clearly indicates that the three CTF exhibit a substantial difference in the conformation of the amyloid core. Our previous study has revealed that full-length TDP-43 and TDP-35 (90–414) shared the same solid-state NMR spectral fingerprint; however, it was not the case for the CTF TDP-16 (267–414). Interestingly, in the present work, we show that shorter CTFs are more prone compared to the larger constructs TDP-43 and TDP-35, to structural polymorphism and to aggregate into distinct structures despite the absence of a few residues from the CTD. For TDP-11, we truncated a part of the GaroS2 domain (residues 400–414) that is not observed by solid-state NMR as the rigid component of the so-called “second core” (i.e., 365–400) by Murray et al. (Fonda et al., 2021). Even though the C-terminal part of the GaroS2 domain (residues 400–414) has been reported neither to be a component of the rigid β-sheet amyloid core of CTF fibrils nor involved in the second core of aged liquid droplets, the truncation of this segment has shown a significant impact on the overall molecular conformation of mature fibrils, as exemplified by TDP-11. Truncation of the C-terminal residues in the TDP-11 construct might destabilize semi-rigid amyloid segments, explaining why more signals are observed in the INEPT experiment of TDP-11 encoding for mobile residues. It reinforces the concept that distinct aggregation-prone domains might interact through weak interaction to prevent their conversion into the cross-β arrangement. Such a hypothesis has been proposed by Fonda *et al.* to explain why in aged liquid droplets, the GaroS2 domain is detected as a rigid β-sheet assembly and not the hydrophobic domain, suggesting a preventive role for GaroS2 to assemble into regular amyloid fibrils and protect the hydrophobic domain from misfolding events (Fonda et al., 2021). Following such a concept, the N-terminal part of the GaroS1 domain may play a similar role as the GaroS2 domain, in that its truncation affects the molecular conformation of mature TDP-43 CTF fibrils and may protect other domains from misfolding events. This highlights the importance of studying the interaction between amyloid and non-amyloid domains in the context of fibril formation.

In line with previous *in vitro* structural studies of TDP-43 and CTD fragments, notably by SSNMR techniques that report on local structural polymorphism, our study reinforces the crucial role of amyloid polymorphism in the pathological context of TDP-43 aggregation. While our *in vitro* aggregation protocol led to the observation of fibrillar objects, previous SSNMR studies based on different purification and aggregation procedures have reported the formation of droplets (without fibrils) or fibrils and droplets (Zhuo et al., 2020; Fonda et al., 2021; Pantoja-Uceda et al., 2021) suggesting complex droplet to fibril transition (Babinchak et al., 2019). The ability of TDP-43 and its CTD fragments to generate alternative structures, undergoing various degree of local polymorphism as detected here by SSNMR, will require further studies to understand the molecular origin of this polymorphism and how it can be interpreted in term of loss-of-function for the native TDP-43 functional fold.

Taken together, our findings strengthen the importance of the low-complexity domains in the aggregation process of TDP-43 and the resulting molecular conformation of mature fibrils. Partial truncation of these domains leads to the generation of distinct fibrillar core arrangements underlying the propensity of TDP-43 to adopt polymorphic amyloid structures under *in vitro* conditions. In particular, the GaroS2 domain, which has not been identified as part of the amyloid core in the high-resolution structure of *ex vivo* TDP-43 fibrils as determined by cryo-EM (Arseni et al., 2022), participates in the rigid amyloid core in our *in vitro* fibril preparation, and constitutes the main structured core of aged liquid droplets (Fonda et al., 2021). It underlines the crucial role of low-complexity domains in the aggregation of pathological amyloid proteins to potentially act as small aggregation-prone segments and constitute an additional source of structural polymorphism.

## 4 Materials and methods

### 4.1 Designing of pET24-TDP expression vectors

All genes encoding TDP constructs have a 6xHis-tag on the N-terminus or the C-terminus and are bordered by NdeI and XhoI restriction sites. These constructs were purchased from Eurofins Genomics. The TDP genes were inserted into the expression vector pET24 (Novagen) by digestion with NdeI and XhoI, followed by ligation using T4 DNA ligase (New England Biolabs).

### 4.2 Recombinant protein expression and purification and aggregation

The TDP proteins were expressed and purified following the same protocol as described in our previous study (Shenoy et al., 2019). In short, the proteins were expressed in *E. coli* strain BL21 (DE3) pLysS transformed with the pET24-TDP vector. The expression and purifications were either in LB or M9 minimal media supplemented ^15^NH_4_Cl and ^13^C-glucose. The bacterial cultures were induced with 0.75 mM IPTG at OD_600_ 0.8 and grown for 3 h 30min at 37°C. The cells were harvested by centrifugation (6000g, 30 min, 4°C) and frozen (−80°C) until purification. Frozen bacterial pellets were thawed and resuspended on ice in 20 ml of lysis buffer (50 mM Tris, 150 mM NaCl, pH 8), sonicated (30 W, 3 × 45 s), and centrifuged at 15,000 g for 1 h at 4°C. The pellet was then resuspended in 20 ml of lysis buffer supplemented with 2% Triton X-100, incubated at 37°C (20 min, 220 r.p.m.), and centrifuged (50,000 g, 10 min, 4°C). To remove the detergent, the pellet was washed with lysis buffer (2 h, 37°C, 220 r.p.m.) and then centrifuged (50,000 g, 10 min, 4°C). The pellet thus obtained was resuspended in 10 ml extraction buffer (50 m Tris, 0.5 m NaCl, pH 8) supplemented with dry guanidine hydrochloride until saturation and incubated overnight (60°C, 220 r.p.m.). The solution was sonicated and centrifuged (250,000 g, 1 h, 16°C). The protein was purified using a 5 ml Histrap HP column with a gradient of 10–500 mM imidazole in denaturing buffer (50 mM Tris, 500 mM NaCl, 8M urea, pH 8). The fraction containing pure protein was pooled and dialyzed two times against 20 mM MES buffer pH 7.5 to remove urea and imidazole and was allowed to self-assemble for 1 week at room temperature. It was then extensively washed in water, centrifuged, and transferred to the ssNMR rotor. The sample quantity per rotor was ∼10 mg. Note that the limited solubility of CTD constructs has hampered further solution NMR analysis of soluble monomers.

### 4.3 Solid-state NMR spectroscopy

The SSNMR experiments were carried out on 600 MHz and 800 MHz ^1^H Larmor frequency spectrometers (Bruker Biospin) at a MAS frequency of 11 kHz and 278K on a triple resonance HCN 3.2 mm MAS probe (Bruker Biospin, Rheinstetten, Germany), using 90 kHz SPINAL-64 ^1^H decoupling. DSS was used as an internal reference. The rigid part of the fibrils was probed using two-dimensional ^13^C–^13^C correlation spectra using a PDSD mixing scheme. A cross-polarization (CP) contact time of 1 ms was used in the initial transfer. Intra-residue correlations were probed using a PDSD mixing time of 50 ms for acquisition times of 20 ms (direct) and 7.5 ms (indirect), 288 scans for TDP-13, 480 scans for TDP-10 and 384 scans for TDP-11 and a recycle delay of 3 s led to a total experimental time of ∼5 days. Sequential correlations were probed using a PDSD mixing time of 150 ms for acquisition times of 20 ms (direct) and 6.3 ms (indirect), 792 scans and a recycle delay of 3 s leading to a total experimental time of ∼7 days. The 2D ^1^H-^13^C INEPT was recorded using acquisition times of 20 and 6 ms in direct and indirect dimensions, respectively, with 128 scans, for a total experimental time of ∼18 h. All two-dimensional spectra were processed using Bruker Topspin with a sine-bell shift of pi/4 and analyzed using CCPNMR (Vranken et al., 2005) and plotted identically using the same contour level (50) and scaling factor (1.18).

### 4.4 X-ray diffraction

Fiber diffraction patterns were measured on a Rigaku FRX rotating anode X-ray generator at 4°C equipped with a Pilatus 200K hybrid pixel detector (DECTRIS Ltd, Baden-Daettwil, Switzerland) at the copper wavelength. The concentrated hydrated samples were mounted in a MicroLoops™ from Mitegen (Ithaca, NY, United States) on a goniometer head under the cold nitrogen flow. Each diffraction pattern corresponds to a 360° rotation along the phi axis with an exposure time of 720 s after subtraction of a ‘blank’ image of the same exposure time with only the loop on the goniometer head.

### 4.5 Transmission electron microscopy

TDP fibrils were diluted twice in 150 mM acetic acid buffer and applied to glow-discharged 300 mesh carbon-coated copper grids for 1 min, washed with water, stained with a 2% uranyl acetate (w/v) solution for 1 min, and dried in dark condition, and monitored in an FEI CM120 transmission electron microscope at an accelerating voltage of 120 kV under TEM low-dose mode at a magnification of 200,00×. TEM images were recorded using a Gatan USC1000 2 k × 2 k camera (Pleasanton, CA, United States).

### 4.6 Attenuated total reflection fourier-transform infrared spectroscopy

The ATR-FTIR spectroscopy was performed on a Nicolet 6700 FT-IR spectrometer (Nicolet Instrument, Madison, WI) equipped with a liquid nitrogen-cooled mercury–cadmium–telluride detector (ThermoFisher Scientific, San Jose, CA, United States), with a spectral resolution of 4 cm^−1^ and a one-level zero filling. The hydrated TDP fibril samples were deposited (10 μl) on a germanium ATR crystal. Excess water was removed with a nitrogen gas flow, and the spectrum was recorded with 200 scans. The spectra were analyzed to determine the secondary structure elements of each protein with an algorithm based on a second-derivative function and a self-deconvolution procedure (GRAMS and OMNIC softwares, ThermoFisher Scientific) to examine the number and wavenumber of individual bands within the spectral range of the amide I band (1700–1,600 cm^-1^).

## Data Availability

The original contributions presented in the study are included in the article/Supplementary Material, further inquiries can be directed to the corresponding authors.
