# Supplementary material for: Structural polymorphism of the low-complexity C-terminal domain of TDP-43 amyloid aggregates revealed by solid-state NMR
Source: Front Mol Biosci. 2023 Mar 29;10:1148302. doi: 10.3389/fmolb.2023.1148302 (PMC10095165; doi:10.3389/fmolb.2023.1148302)
Supplement: Supplementary file 1 [file Table1.DOCX]

Supporting Information

Structural polymorphism of the low-complexity C-terminal domain of TDP-43 amyloid aggregates revealed by solid-state NMR

Jayakrishna Shenoy K.P.^1^, Alons Lends^1^, Mélanie Berbon^1^, Muhammed Bilal^1^, Nadia El Mammeri^1^, Mathilde Bertoni^1^, Ahmad Saad^1^, Estelle Morvan^2^, Axelle Grélard^1^, Sophie Lecomte^1^, François-Xavier Theillet^3^, Alexander K. Buell^4^, Brice Kauffmann^2^, Birgit Habenstein^1^*, Antoine Loquet^1^*


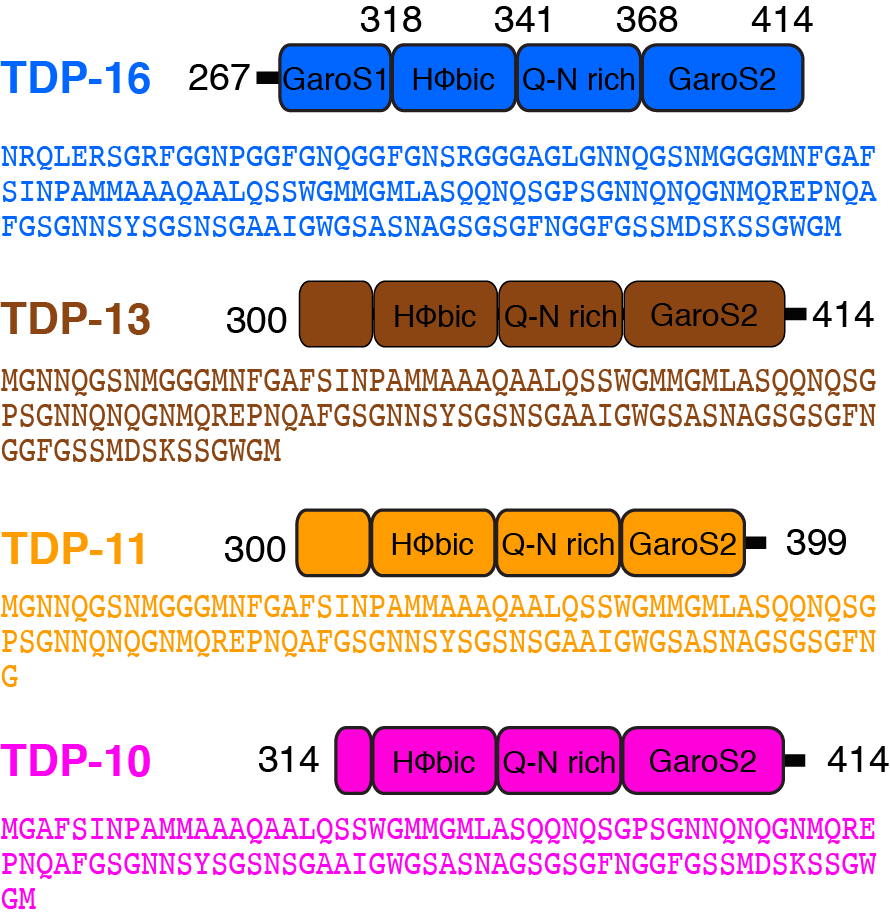


**Supplementary Figure 1:** Primary sequences of TDP constructs. The initial Methionine residues(black) in the sequences is from the construct and not from TDP-43.


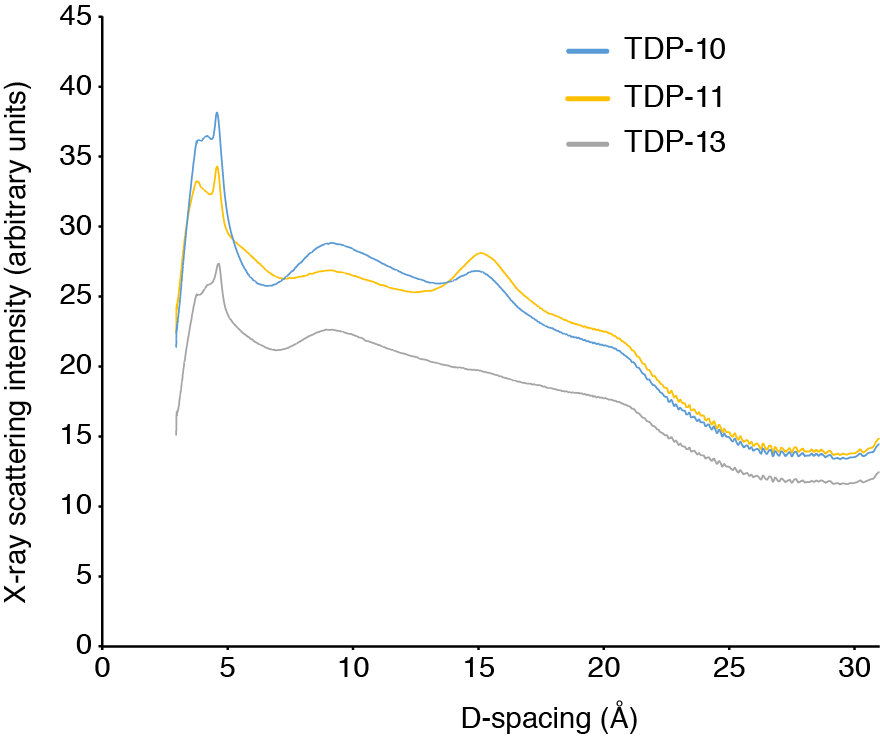


**Supplementary Figure 2:** Diffraction intensity plot of TDP-10, TDP-11 and TDP-13 amyloid fibrils.


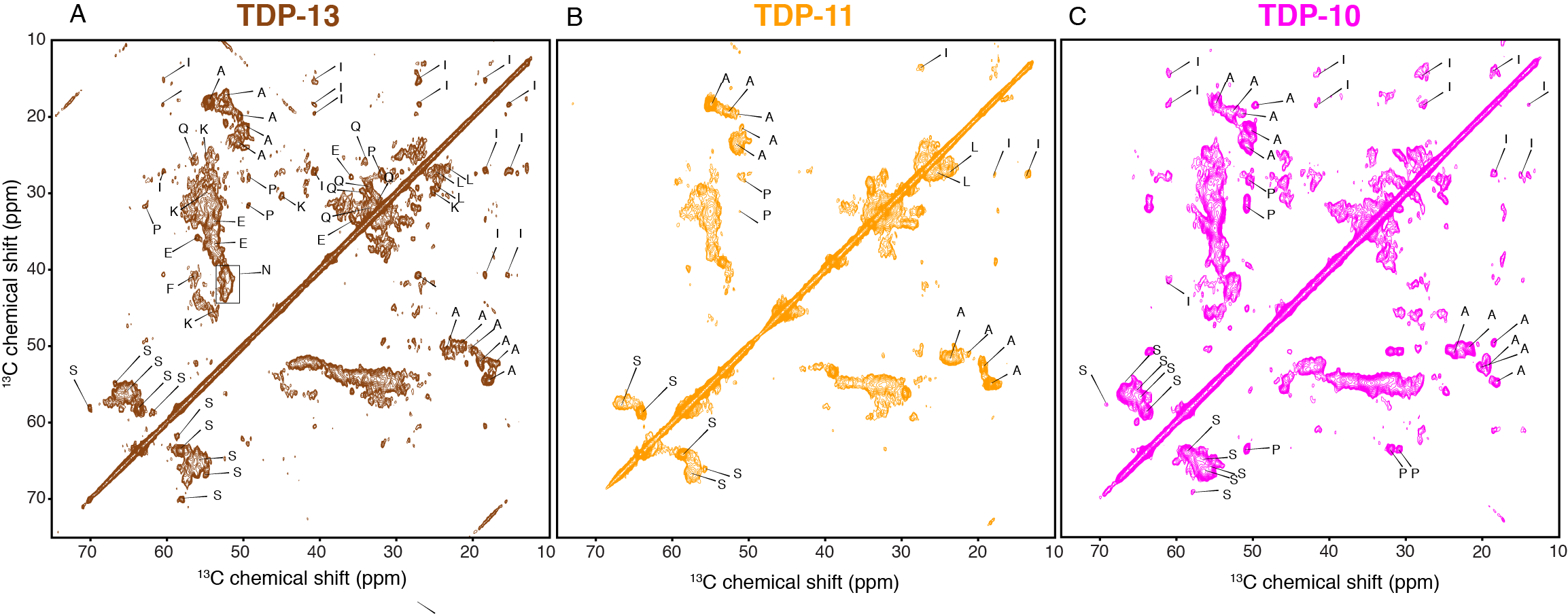


**Supplementary Figure 3: Characterization of rigid residues in TDP-43 CTF aggregates.** ^13^C-^13^C PDSD experiments of TDP-13 (in brown) (A), TDP-11(in orange) (B), and TDP-10 (in pink) (C) amyloid aggregates. The data were recorded at a ^1^H frequency of 800 MHz and 600MHz at 11 kHz MAS at 278 K, using a mixing time of 50 ms to reveal intra-residue correlations.
