# Supplementary material for: Structural polymorphism of the low-complexity C-terminal domain of TDP-43 amyloid aggregates revealed by solid-state NMR
Source: Front Mol Biosci. 2023 Mar 29;10:1148302. doi: 10.3389/fmolb.2023.1148302 (PMC10095165; doi:10.3389/fmolb.2023.1148302)
Supplement: Supplementary file 2 [file Table2.DOCX]

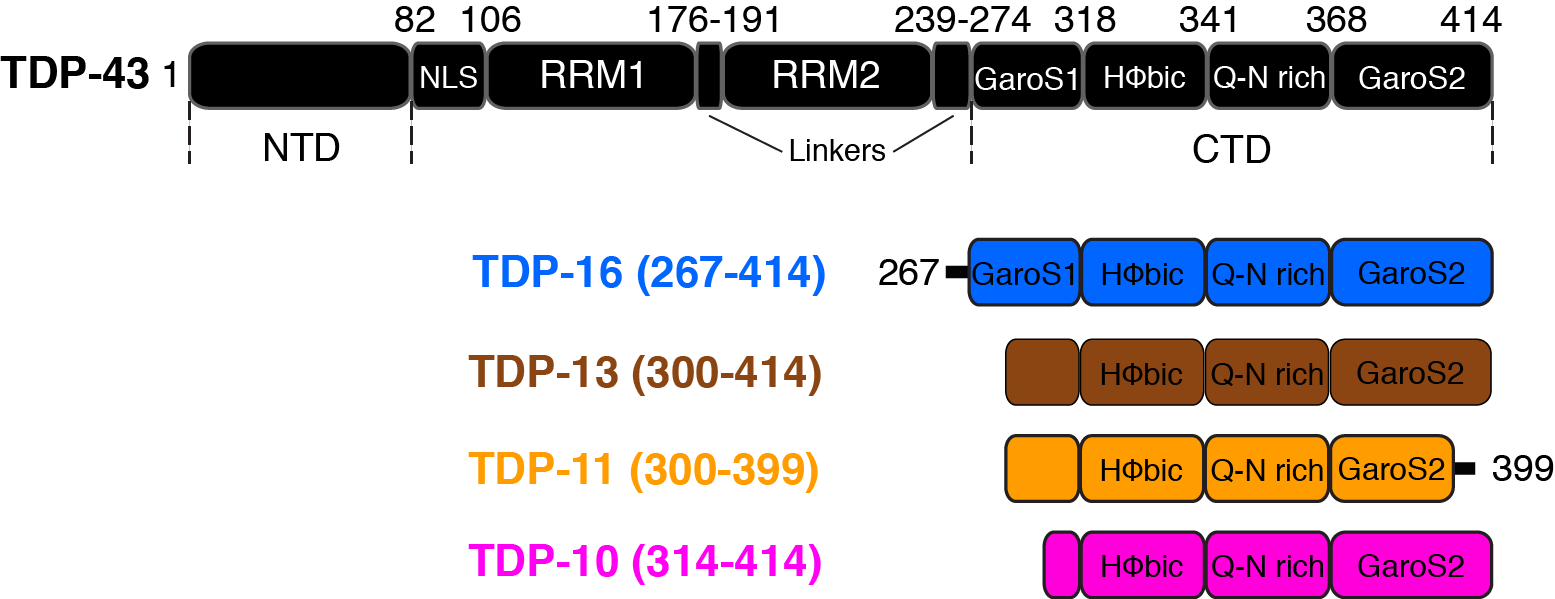


**Figure 1**: **Sequences of TDP-43 and various low-complexity C-terminal domain fragments used in this study.** Boxes are used to denote different functional/structural domains. The nomenclature of the different CTD fragments is chosen to be consistent with ^33,75^.


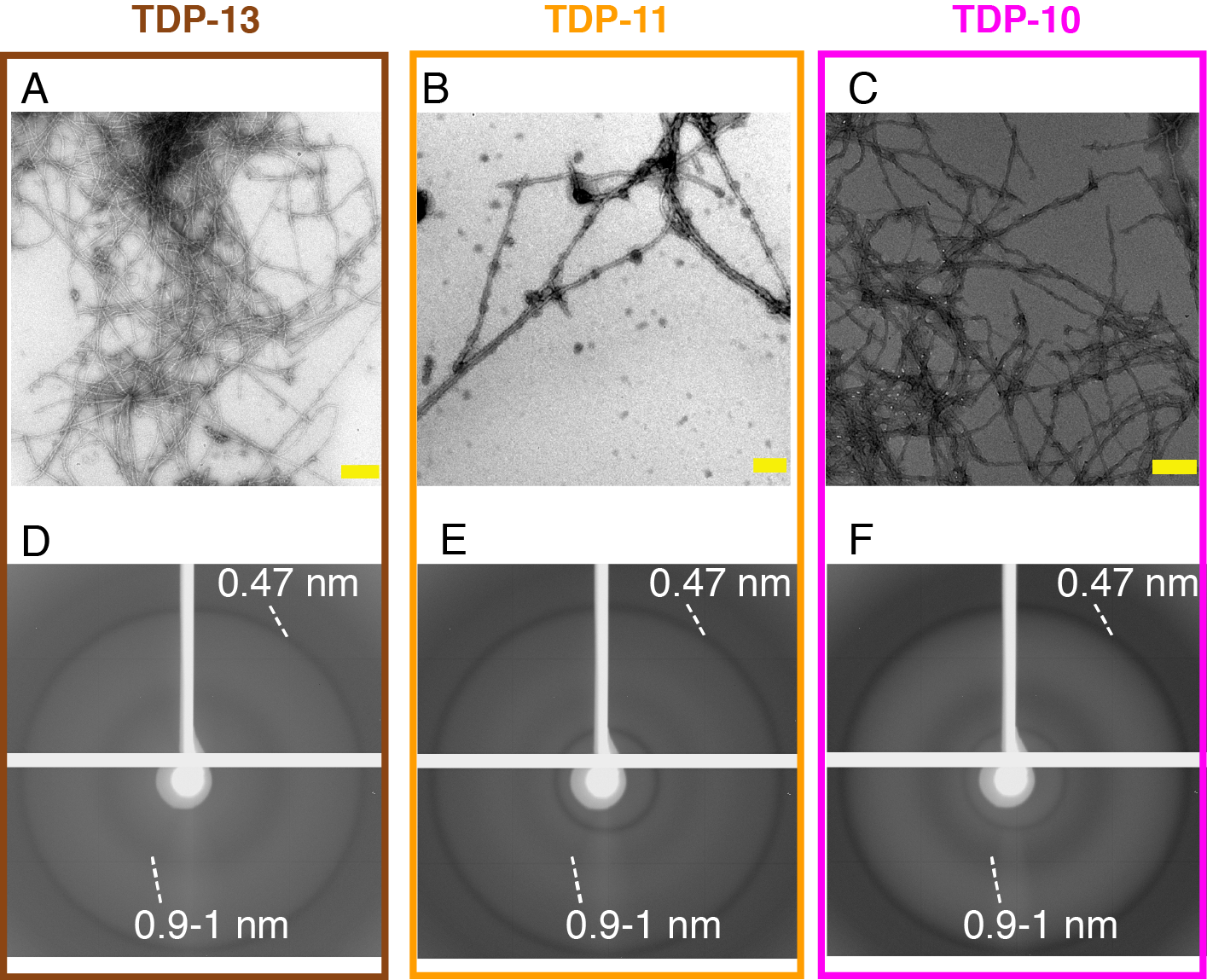


**Figure 2**: **Structural analysis of low complexity CTFs aggregates.** (A-C) Negatively stained electron micrographs of TDP-13 (A), TDP-11 (B), and TDP-10 (C) aggregates. Scale bars (in yellow) are 200 nm. (D-F) X-ray diffraction patterns of TDP-13 (D), TDP-11 (E), and TDP-10 (F) aggregate display characteristic cross-β reflections. Reflections at 4.7 Å and 9-10 Å are highlighted.


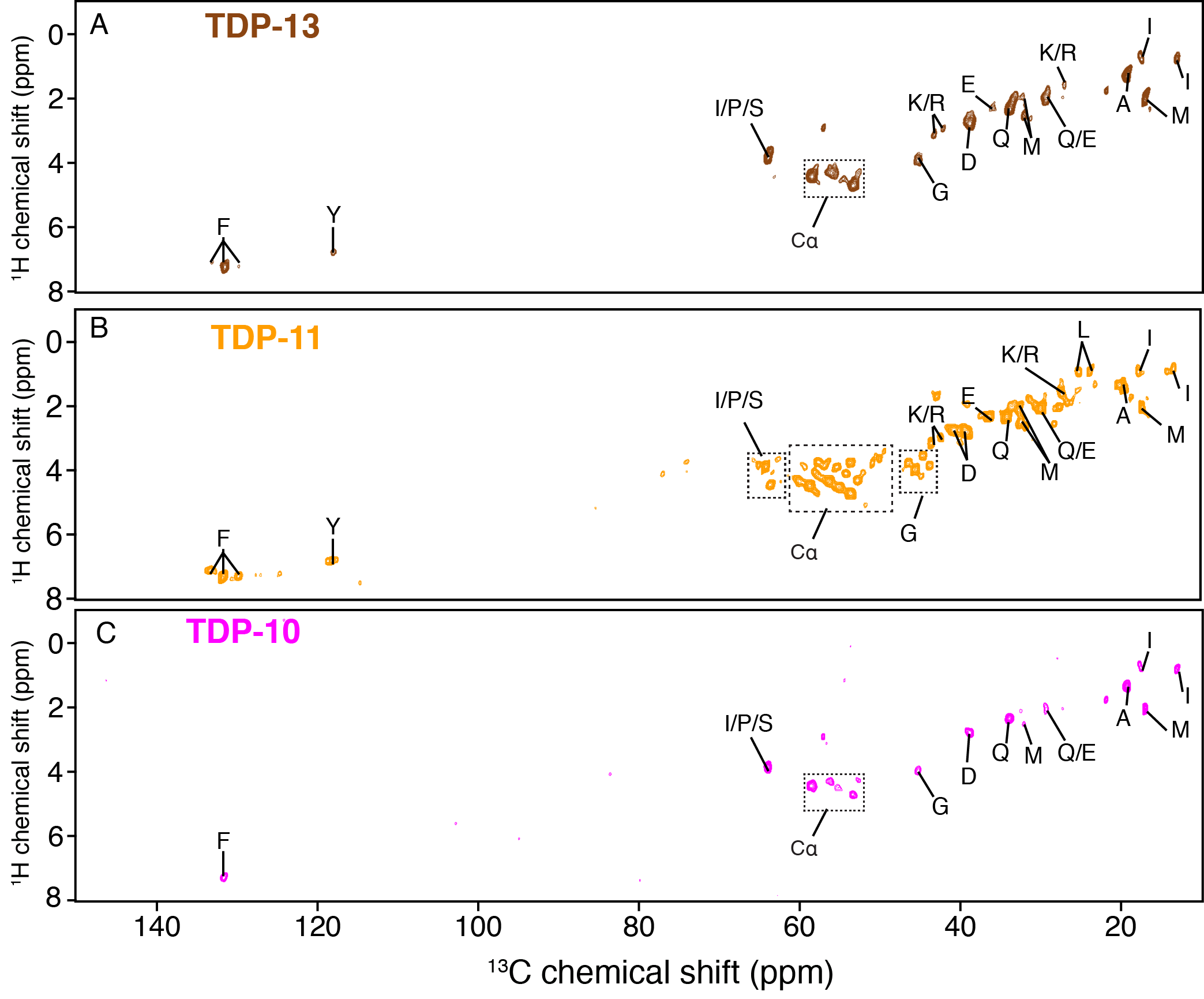


**Figure 3**: **Detection of protein segments associated with high mobility by MAS NMR.** ^1^H-^13^C INEPT experiments of TDP-13 (in brown) (A), TDP-11(in orange) (B), and TDP-10 (in pink) (C) amyloid aggregates. The data were recorded at a ^1^H frequency of 600 MHz, 11 kHz MAS at 278 K.


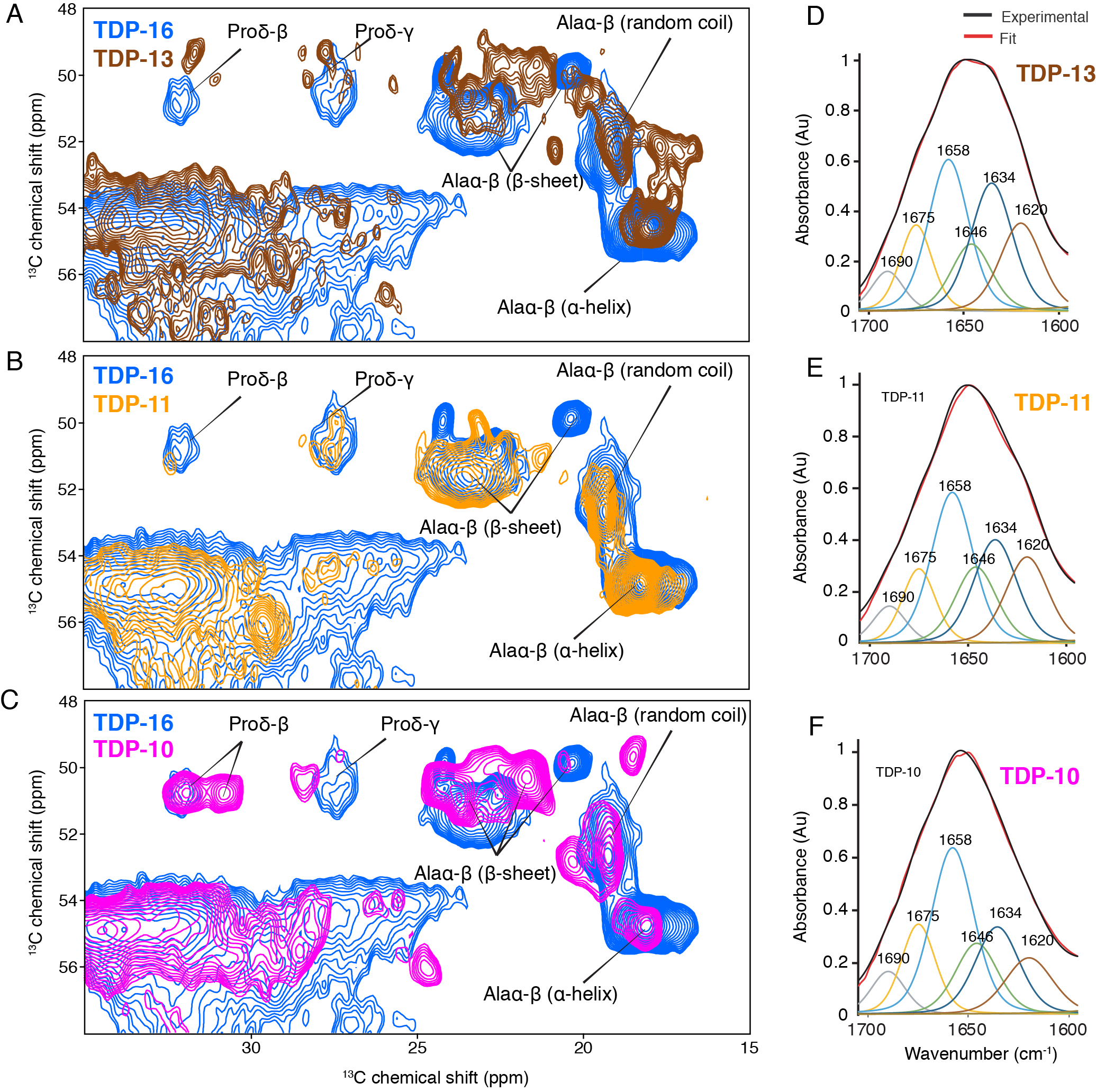


**Figure 4. Comparison of the rigid cores of TDP-10, TDP11, and TDP-13 CTF aggregates by MAS NMR and FT-IR spectroscopy.** (A-C) Spectral excerpts from the overlay of 2D ^13^C-^13^C PDSD spectra (mixing time of 50 ms) of (A) TDP-13 (brown), (B) TDP-11 (orange), and (C) TDP-10 (pink) compared with TDP-16 (blue). Spectra were recorded at 600 MHz, 11 kHz MAS frequency at 278 K. Alanine Cα–Cβ and proline Cδ–Cβ correlations with chemical shift-dependent secondary structure are marked. FT-IR spectra of (D) TDP-13, (E) TDP-11, and (F) TDP-10 aggregates in the amide I and II range, displaying the experimental (black) and fitted curve (red). The deconvolution of amide bands are shown on the FTIR spectra, displaying contribution of secondary structure elements, namely parallel and antiparallel β-sheet: 1634 cm^−1^ (dark blue), 1620 cm^−1^ (brown), and 1690 cm^−1^ (grey); α-helix: 1658 cm^−1^ (sky blue); turns: 1675 cm^−1^ (yellow), and random coil: 1646 cm^−1^ (green).

|  |  | Percentage of structural elements (%) | | | |
| --- | --- | --- | --- | --- | --- |
| Secondary structure element | Wavenumbers (cm^-1^) | TDP-43 | TDP-13 | TDP-11 | TDP-10 |
| β-sheets // or anti // | 1620, 1635, 1690 | 52 | 46 | 42 | 49  13  26  12 |
| Random coil | 1646 | 15 | 13 | 18 |  |
| α−helices | 1658 | 23 | 28 | 28 |  |
| Turn | 1675 | 10 | 13 | 12 |  |
|  |  |  |  |  |  |

**Table 1**: Secondary structure content of TDP-43 CTF amyloid fibrils probed by ATR‐FTIR analysis


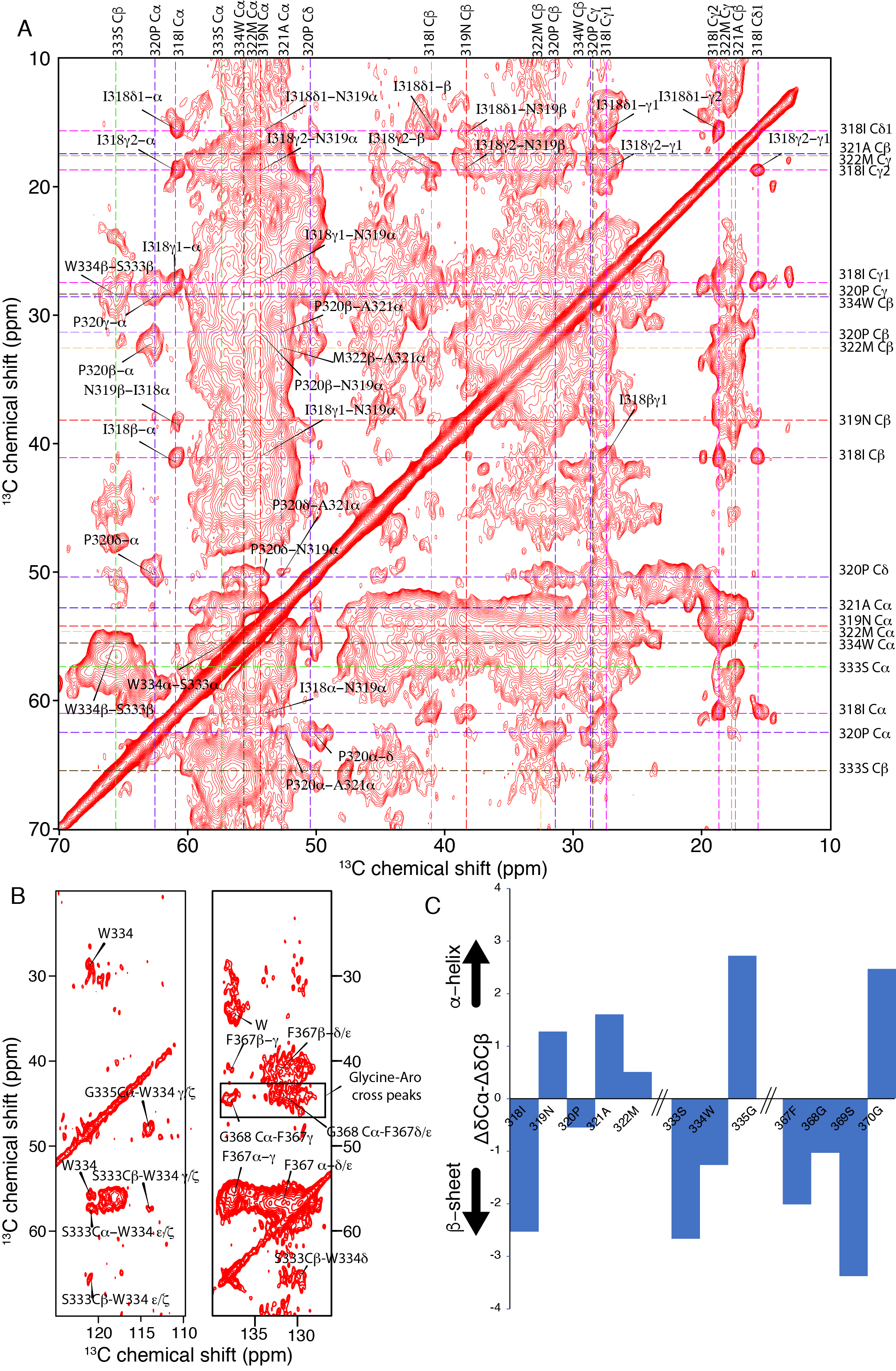


**Figure 5** Investigation of the amyloid core of TDP-13 amyloid aggregates. (A) Aliphatic and (B) aromatic region of 2D ^13^C-^13^C correlation experiment of TDP-13 amyloid aggregates recorded at a ^1^H frequency of 800 MHz at 278 K and 11 kHz MAS. A PDSD mixing time of 150 ms was used to favor sequential (i.e., residue i to i ± 1) connectivity indicated by color-coded dotted lines. (C) The secondary chemical shift analysis of stretches 318–322, 333–335, and 367-370 comparing experimentally obtained chemical shift with that of random-coil conformation using the equation ΔδCα–ΔδCβ ^95^. Positive and negative values corresponds to α-helical or β-strand conformation, respectively.
